# Probiotic modulation of maternal gut and milk microbiota and potential implications for infant microbial development in the perinatal period

**DOI:** 10.3389/fcimb.2025.1715989

**Published:** 2025-12-11

**Authors:** Kian Deng Tye, XiaoYi Liu, Chan Huang, Chen Li, ChaoLi Wu, JunLue Lin, YongJin Yu, XinZi Lin

**Affiliations:** 1 Department of Obstetrics, The Fifth Affiliated Hospital of Guangzhou Medical University, Guangzhou, China; 2 Department of Neonatology, The First Affiliated Hospital of Jinan University, Guangzhou, China

**Keywords:** probiotics, maternal microbiota, immune programming, microbiome modulation, reproductive immunology

## Abstract

**Background:**

Probiotics are live microorganisms that may enhance or restore gut microbiota. They are often recommended during pregnancy and infancy for potential benefits, but evidence is inconclusive. This study aimed to investigate probiotic supplementation’s effects on maternal and infant gut and milk microbiota and its link to nutrient intake during pregnancy.

**Method:**

A total of 23 pregnant women were enrolled and divided into a probiotic group (n = 11) and a non-probiotic control group (n=12). Probiotic effects were evaluated through fecal and milk microbiota analysis via 16S rRNA gene sequencing. Nutrient intake data were collected to assess differences linked to probiotics. Key microbiota diversity and richness were analyzed using linear discriminant analysis effect size (LEfSe) and weighted gene co-expression network analysis (WGCNA) to explore associations with diet and sample characteristics. Predictive microbial pathway characteristics were identified using time series analysis, random forest algorithms, and logistic regression models.

**Results:**

Nutrient intake did not significantly differ between groups, and overall microbial diversity and richness were stable. However, LEfSe revealed distinct genera in both maternal gut and milk microbiota linked to probiotic intake. WGCNA identified microbial modules correlated with specific nutrient patterns and sampling conditions. Predictive genus clusters associated with probiotics demonstrated robust classification performance, suggesting functional shifts in microbial communities with potential implications for immune programming in early life.

**Conclusion:**

Probiotic supplementation during pregnancy may modulate key microbial taxa in maternal gut and milk, potentially influencing microbial recognition and immune signaling in the maternal–infant dyad. These findings highlight complex diet–microbiota–immune interactions within reproductive and lactational systems, offering insights into strategies for enhancing maternal and neonatal health resilience.

## 1 Introduction

During pregnancy, microbiota in different bodily locations, including the gut, vagina, oral cavity, and breast, undergo apparent changes as the fetus grows (Backhed et al., 2015; Nuriel-Ohayon et al., 2016; Cui et al., 2018; Bohan et al., 2019). These alterations might influence the maternal metabolic profile and contribute to the microbial population of the infant (Dominguez-Bello et al., 2010). Consequently, the role of the microbiome in pregnancy has become a subject of considerable interest. The maternal microbiota from the third trimester of normal pregnancy has been reported to show signs of inflammation, adiposity, and insulin insensitivity similar to those observed in obesity (Gohir et al., 2015). Likewise, it has been verified that the placenta has a unique microbiome associated with preterm birth, indicating that cross-talk between bacterial communities and pregnant women may be necessary (Gohir et al., 2015; Sanders, 2016; Shahriari et al., 2021). Many factors can influence the microbiome structure. Dietary intake, physical exercise, and probiotic supplementation are critical factors. Diet is one of the principal determinants of gut microbiota composition (Gohir et al., 2015; Sanders, 2016; Heiss and Olofsson, 2019; Shahriari et al., 2021). The structure of the human diet is changing, and the composition and structure of gut microbiota differ among different people. Diet has been reported to be a non-negligible factor in changes in gut microbiome structure during pregnancy (Gohir et al., 2015). Probiotics, defined as live microorganisms conferring health benefits when administered in adequate amounts, have been proposed but not conclusively proven to beneficially modulate microbiota composition and host metabolism (Houghteling and Walker, 2015). Mechanistic studies suggest that probiotics can influence immune maturation and metabolic pathways through short-chain fatty acid (SCFA) production, epithelial barrier modulation, and cytokine regulation (Hui et al., 2021). Interventional trials have shown associations between maternal probiotic use and reduced risks of gestational diabetes, allergic outcomes, or eczema in infants, though results remain heterogeneous and context-dependent (Jimenez et al., 2008; Hyman et al., 2014; Jie et al., 2017). Recent research has shown that the human gut microbiota is primarily established before the age of 2 years and may influence neurodevelopment and immune programming (Dominguez-Bello et al., 2010). Neuroregulatory signals generated by intestinal microbes can shape neuronal function and behavior during critical developmental windows. This supports the hypothesis that the fetal–infant period may be a key window for microbiota imprinting (Koren et al., 2012; Mahdizade Ari et al., 2022). Based on these concepts, we proposed the “gut microbiota fetal origin hypothesis,” which posits that maternal microbiome management from pregnancy through postpartum stages may influence infant microbiota establishment and, potentially, future health outcomes. To explore this hypothesis, we recruited 23 pregnant women divided into a probiotic intake group (n=11) and a non-probiotic intake group (n=12). We investigated the association between probiotic intake and maternal diet, followed by 16S rRNA sequencing of maternal gut microbiota at 32–34 and 40 gestational weeks, as well as corresponding infant microbiomes at delivery, 3 days, 14 days, and 6 months post-birth. Using weighted gene co-expression network analysis (WGCNA), we identified microbiota modules correlated with maternal and infant dietary profiles. The modules most relevant to the neonatal stage were analyzed through time-series clustering (mfuzz), followed by random forest modeling to identify putative microbial biomarkers. Functional pathway prediction via PICRUSt2 was performed to explore the potential metabolic functions associated with early microbial colonization. Based on these observations, we propose a ‘fetal origin hypothesis’ of gut microbiota development in this study.

## 2 Materials and methods

This study was conducted per Helsinki guidelines and approved by the Fifth Affiliated Hospital Ethics Committee of Guangzhou Medical University (approval number: KY01-2023-10-04). All participants provided written informed consent before beginning the study.

### 2.1 Study design and sample collection

This study recruited 23 pregnant women scheduled to give birth at the Fifth Affiliated Hospital of Guangzhou Medical University between 2022 and 2023 who met all inclusion criteria. Informed consent was obtained from all participants before 32 weeks of gestation, and the women were followed through pregnancy until 6 months post-delivery. The 23 pregnant women were categorized into two groups based on their probiotic intake: a probiotic intake group (P group) and a non-probiotic intake control group denoted as U group. This study was conducted as an open-label, unblinded intervention, as the control group did not receive a placebo. Participants and investigators were aware of group allocation, which may influence subjective measures such as self-reported dietary intake. The P group began taking a standard probiotic formulation containing live strains of *Bifidobacterium longum* (≥5×10^6^ CFU per tablet), *Lactobacillus bulgaricus* (≥5×10^5^ CFU per tablet), and *Streptococcus thermophilus* (≥5×10^5^ CFU per tablet), supplied as triple-viable tablets (Inner Mongolia Shuangqi Pharmaceutical Co., Ltd). Supplementation started at 32–34 gestational weeks, with participants consuming two tablets twice daily (total daily dose: *B. longum* ≥2 × 10^7^ CFU, *L. bulgaricus* ≥2 × 10^6^ CFU, *S. thermophilus* ≥2×10^6^ CFU). The prescribed dosage was two tablets twice daily, and supplementation continued until 40 gestational weeks. In contrast, the U group did not receive any probiotics. Fecal samples from both groups were collected at the start of the study, between 32 and 34 gestational weeks. Breast milk samples were then collected from both groups 6 months after delivery. Sampling time-points (32–34 weeks, 40 gestational weeks (term), 3 days, 14 days, and 6 months) were chosen to capture key physiological windows of maternal metabolic adaptation and infant microbial succession. All participants completed a comprehensive Food Frequency Questionnaire (FFQ) with one-on-one guidance upon admission to the hospital for delivery, ensuring accurate dietary intake records. Sampling time-points (32–34 weeks, delivery, 3 days, 14 days, and 6 months) were chosen to capture key physiological windows of maternal metabolic adaptation and infant microbial succession. To simplify data analysis, groups and sample codes were assigned according to sample type, group, and collection time, as summarized in **Table 1**. Samples were transferred to the laboratory within 2 hours in insulated boxes containing dry ice and stabilized using OMNIgene-GUT collection buffer to maintain microbial viability prior to −80°C storage. Groups were matched for maternal age, pre-pregnancy BMI, parity, and socioeconomic status, with no significant baseline differences observed (*p* > 0.05).

**Table 1:** Group names and descriptions of all samples.

Group name	Description	Number
UPMF	Fecal samples of U group at 32–34 gestational weeks	12
PPMF	Fecal samples of P group at 32–34 gestational weeks	11
UPLF	Fecal samples of U group at 40 weeks (term)	12
PPLF	Fecal samples of P group at 40 weeks (term)	11
URZ6	Milk samples of U group at 6 months after birth	8
PRZ6	Milk samples of P group at 6 months after birth	6
U1BF	Fecal samples at birth for U group infants	12
U2BF	Fecal samples at 3 days after birth for U group infants	12
U3BF	Fecal samples at 14 days after birth for U group infants	12
UDB6	Fecal samples at 6 months after birth for U group infants	12
P1BF	Fecal samples at birth for P group infants	11
P2BF	Fecal samples at 3 days after birth for P group infants	11
P3BF	Fecal samples at 14 days after birth for P group infants	11
PDB6	Fecal samples at 6 months after birth for P group infants	11

### 2.2 Participant inclusion and exclusion criteria

#### 2.2.1 Pregnant women

Eligible participants were Chinese women experiencing their first pregnancy and at term (≥37 weeks gestation). Exclusion criteria included a history of gastrointestinal diseases or a family history of gastrointestinal issues, vaginitis, antibiotic use, hypertension, diabetes mellitus, hyperthyroidism, rheumatic or other autoimmune diseases, endocrine or metabolic disorders, pregnancy-specific complications, blood transfusion, organ transplantation, or immunotherapy. Enrollment and exclusion procedures are detailed in **Supplementary Figure S1**.

#### 2.2.2 Infants

Eligible infants were born full-term (≥37 weeks gestation) with a weight above 2500 g. Exclusion criteria for infants included low birth weight (<2500 g), severe complications during delivery, or the presence of congenital diseases. This design allowed for a controlled examination of the effects of probiotic supplementation on maternal and infant gut microbiota composition and diversity over time.

### 2.3 Genomic analysis

Total DNA was isolated from all samples (approximately 0.5 g each) using a standard cetyltrimethylammonium bromide (CTAB) DNA extraction protocol to ensure high-quality yield. DNA integrity and quality were assessed on a Tapestation 4200 system (Agilent Technologies, Palo Alto, CA, USA). In contrast, DNA concentration was quantified using a Qubit dsDNA HS (high sensitivity) kit (Cat. No. Q32851, Life Technologies, Carlsbad, CA, USA). For the amplification of the 16S rRNA genes, an Ion Torrent 16S Metagenomics Kit (Cat. No. A26216, Thermo Fisher Scientific, MA, USA) was employed. The Ion Torrent 16S Metagenomics Kit targets V2–V9 regions to maximize phylogenetic breadth across bacterial phyla, although this design may slightly reduce taxonomic resolution compared with single-region amplicons (e.g., V3–V4). Following the manufacturer’s protocol, PCR amplification was performed in two pools, each using primer sets targeting specific hypervariable regions of the 16S rRNA gene. Pool 1 contained primers for the V2, V4, and V8 regions, while Pool 2 targeted the V3, V6-7, and V9 regions. The amplicon lengths for each hypervariable region were as follows: V2 (250 bp), V3 (215 bp), V4 (288 bp), V6-7 (260 bp), V8 (295 bp), and V9 (209 bp). After amplification, DNA concentration was quantified using the Qubit dsDNA HS (High Sensitivity) Assay Kit. (Thermo Fisher Scientific). Library preparation was conducted with the Ion Plus Fragment Library Kit (Cat No. 4471252, Thermo Fisher Scientific) and the Ion Xpress Barcode Adapters 1–16 Kit (Cat. No. 4471250, Thermo Fisher Scientific), adhering strictly to the manufacturer’s protocol. Post-PCR, libraries were purified, and concentrations were measured on the Tapestation 4200. Sequencing was performed on an Ion S5TM XL platform to achieve comprehensive genomic profiling. Of the 138 samples processed, 134 (97.1%) passed quality control, while four infant fecal samples failed sequencing and were excluded from downstream analyses. To validate extraction performance, CTAB efficiency was benchmarked against the QIAamp DNA Stool Mini Kit in six randomly selected samples, demonstrating comparable microbial yield and composition (Pearson r = 0.93, *p* < 0.01).

### 2.4 Analysis of 16S rRNA gene sequencing data

Raw sequencing data were initially processed by splitting, assembling, and merging reads using the FLASh tool (version 1.2.11) to improve read continuity. Quality filtering was performed through the QIIME2 suite to ensure high-quality reads. Chimera removal was achieved using the UCHIME algorithm, which retained only qualified reads for subsequent analysis. These reads were clustered into Operational Taxonomic Units (OTUs) at a 97% similarity threshold by employing USEARCH (version 11), and taxonomic information was annotated using the Silva database (version 132). Homopolymer error correction was applied using Ion Reporter v5.18, followed by denoising through DADA2 within QIIME2 to minimize Ion Torrent–specific sequencing errors. Alpha diversity, a measure of species richness and evenness within each sample, was assessed using six indices: observed species, Chao1, Shannon, Simpson, ACE, and Good’s coverage, all computed within QIIME2. Beta diversity analysis, which examines the variation in species composition across samples, was conducted based on weighted and unweighted UniFrac distances in QIIME2. Batch effects arising from sequencing runs and sample types were corrected using the ComBat function in the sva R package (v3.46.0), ensuring that observed microbial differences reflected biological rather than technical variation. For visualization of sample clustering patterns, Principal Coordinates Analysis (PCoA) was conducted using the R package FactoMineR (version 2.8) with ggplot2 (version 3.4.3) for graphical output, enabling a detailed examination of species complexity differences among samples.

### 2.5 Weighted gene co-expression network analysis

To explore relationships between microbiota and their combined impact on follow-up data and maternal and infant groups, Weighted Gene Co-Expression Network Analysis (WGCNA) was applied. Given the small cohort (n = 23), WGCNA and machine-learning analyses were performed in an exploratory manner. Module detection robustness was assessed through repeated subsampling, and random-forest models were internally validated by five-fold cross-validation to minimize overfitting. Prior to WGCNA, OTU counts were normalized using a centered log-ratio transformation, low-variance OTUs (< 0.1) were excluded, and the soft-thresholding power (β = 6) was chosen based on the scale-free topology criterion (R² > 0.85). This technique constructed a co-occurrence network of OTUs based on all normalized OTU counts. The resulting network was divided into several modules, each representing clusters of closely related OTUs. Correlations were then calculated between each module, the follow-up data, and maternal and infant groups. Modules most relevant to groups U1BF and P1BF were selected for time series analysis, conducted using the R package mfuzz (version 2.6.0). OTUs within the highest abundance clusters were subjected to function enrichment analysis using PICRUSt2 (version 2.5.2) to predict potential functional pathways mapped to the Kyoto Encyclopedia of Genes and Genomes (KEGG) database. Random-forest models employed a 70/30 training-testing split with five-fold cross-validation, and feature importance was determined via 1,000 permutations. These clusters were further analyzed with random forest and logistic regression to identify potential biomarkers at the genus level. Random forest modeling was carried out using the R packages randomForest (version 4.7) and pROC (version 1.18.4) to assess prediction accuracy.

### 2.6 Statistical analysis

All statistical analyses were performed using R software (version 4.3.1) and QIIME2 (version 2023.5). To control for false discoveries, *p*-values from multiple comparisons were adjusted using the Benjamini–Hochberg false discovery rate (FDR) method, with *adjusted p* < 0.05 considered statistically significant. Differences in alpha diversity indices, clinical parameters, and dietary variables between groups were analyzed using the Wilcoxon rank-sum test, independent-samples *t*-test, Chi-square test, or Fisher’s exact test, as appropriate. Principal Coordinates Analysis (PCoA) and Analysis of Similarities (ANOSIM) based on weighted and unweighted UniFrac distances (999 permutations) were applied to assess inter- versus intra-group beta diversity. Taxonomic differences across groups were evaluated by the Kruskal–Wallis H test, and differential microbial features were identified using Linear Discriminant Analysis Effect Size (LEfSe) with a minimum LDA score > 2.0 and FDR-adjusted *p* < 0.05 to ensure effect-size reliability. To account for repeated measures and longitudinal sampling, generalized linear mixed models (GLMM) were implemented using the MaAsLin2 package (v1.10.0), with subject ID treated as a random effect and time point as a fixed factor. Associations between microbial taxa and clinical or dietary parameters were further validated using Spearman’s rank correlation corrected for multiple testing. For machine-learning analyses, random forest classifiers were trained with a 70/30 training–testing split and five-fold cross-validation to mitigate overfitting. Feature importance was estimated via 1,000 permutations. All plots were generated using ggplot2 (v3.4.3).

## 3 Results

### 3.1 Effect of probiotic supplementation on dietary intake in pregnancy

To investigate the impact of probiotic supplementation on dietary intake during pregnancy, we developed a questionnaire based on the 2016 Dietary Guidelines for Chinese Residents (Wang et al., 2016). The questionnaire included 79 items covering various food categories such as grains, legumes, vegetables, fruits, meats, eggs, dairy products, mushrooms, nuts, and beverages. We collected daily, weekly, monthly, and annually data on food intake frequency for each enrolled pregnant woman, calculating nutrient intake based on the Chinese Food Ingredient List. Nutrient terms analyzed included daily energy intake (kcal), protein, carbohydrates, dietary fiber, monounsaturated fatty acids (MUFA), polyunsaturated fatty acids (PUFA), vitamins A, B1, B2, B3, C, E, calcium (Ca), magnesium (Mg), iron (Fe), zinc (Zn), and selenium (Se). Statistical analysis revealed no significant differences in nutrient intake between the probiotic and non-probiotic groups. Analysis of probiotic taxa confirmed successful detection in fecal samples of supplemented mothers and infants. Bifidobacterium longum relative abundance was significantly higher in the probiotic group compared with controls at all measured time points (maternal: 3.2% ± 0.8% vs. 1.1% ± 0.5%, p < 0.001; infant: 2.5% ± 0.7% vs. 0.9% ± 0.4%, p < 0.001). Lactobacillus bulgaricus was detected at low but measurable levels in supplemented participants (maternal: 0.9% ± 0.3%; infant: 0.6% ± 0.2%) and was absent or negligible in control samples. These results confirm compliance and effective transient colonization of the administered probiotics.

### 3.2 Overview of the 16S rRNA gene sequencing data

**Figure 1:**
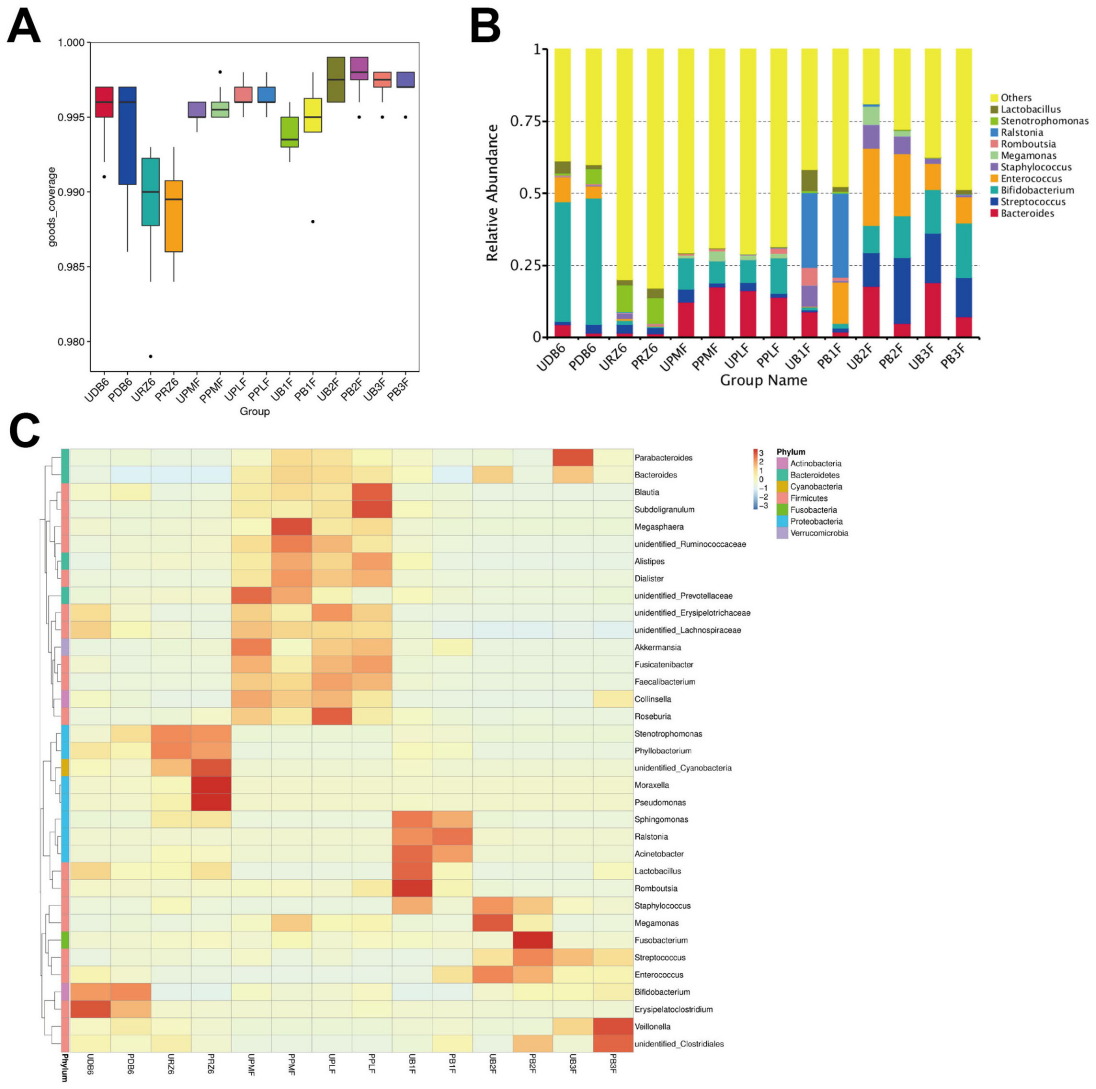
Overview of the 16S rRNA gene sequencing data. **(A)** Box plot showing Good’s coverage of each group. **(B)** The heat map shows the average operational taxonomic unit (OTU) abundance of each group at the genus level. **(C)** Relative abundance of the top 10 OTUs in each group at the genus level.

12,006,319 sequences were sorted into 9,538 operational taxonomic units (OTUs) based on taxonomic classification. The Good’s coverage indices for observed OTUs, as shown in **Figure 1A**, averaged 0.995 across all groups, confirming thorough sampling. A heatmap illustrating the taxonomic data for all participants is provided in **Figure 1B**, while relative abundance of the top 10 genera in each group is shown in **Figure 1C**.

### 3.3 Richness and diversity analysis of microbiota

**Figure 2:**
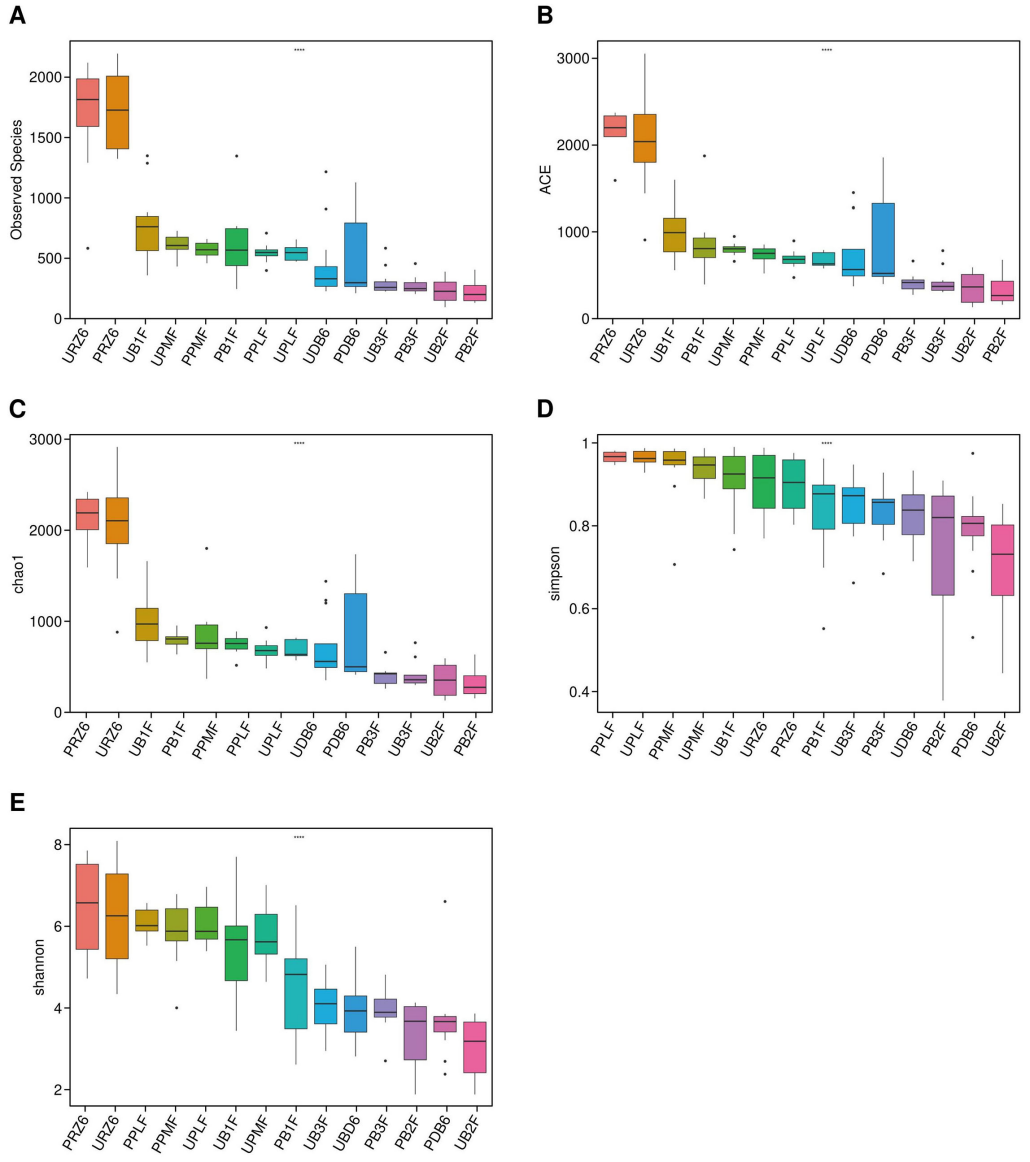
Alpha diversity metrics across all groups. **(A–E)** Box plots of observed species, ACE, Chao1, Shannon, and Simpson indices in all groups, respectively.

**Figure 3:**
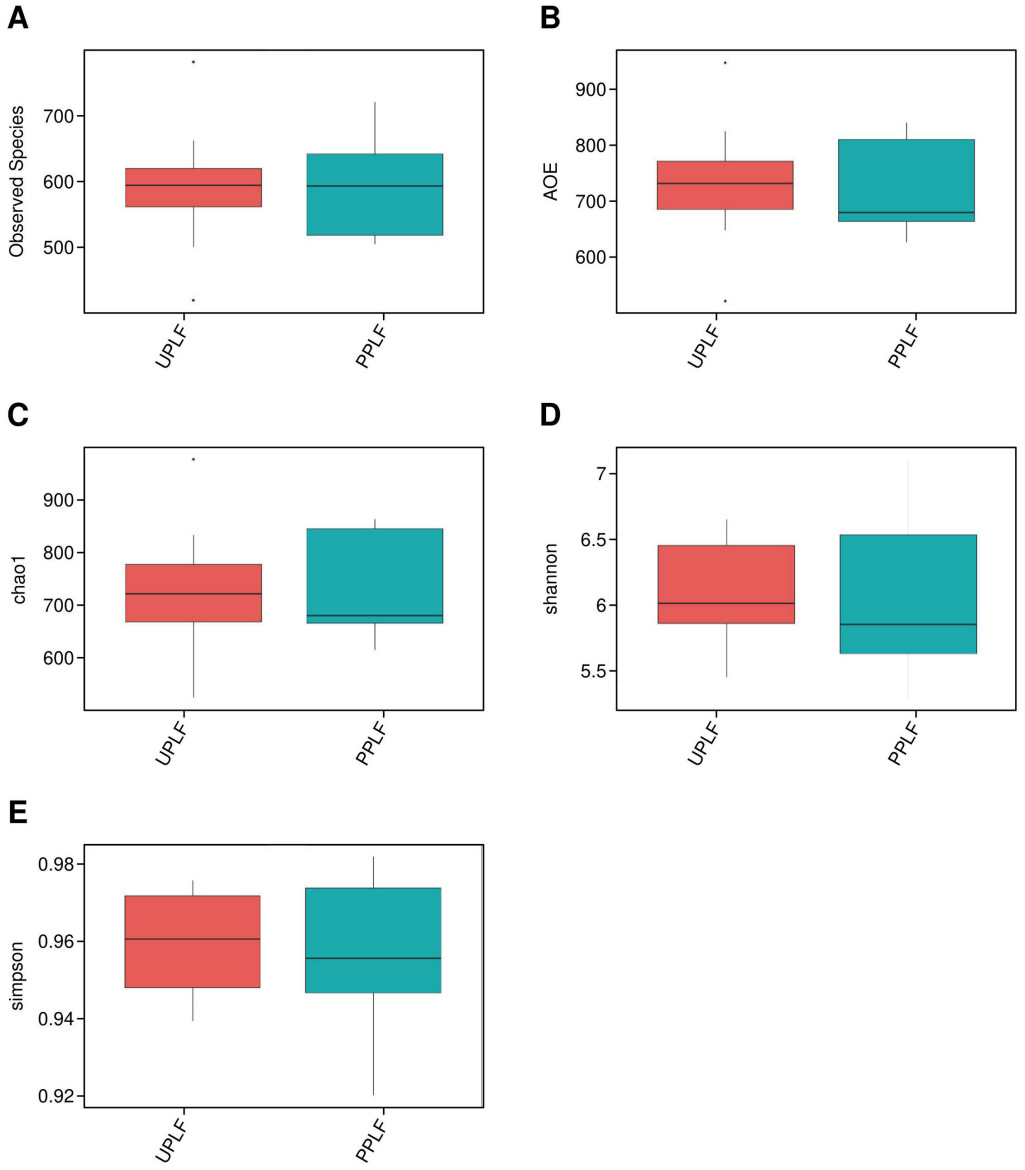
Alpha diversity comparison between UPLF and PPLF groups. **(A–E)** Box plots of observed species, ACE, Chao1, Shannon, and Simpson indices in all groups, respectively.

**Figure 4:**
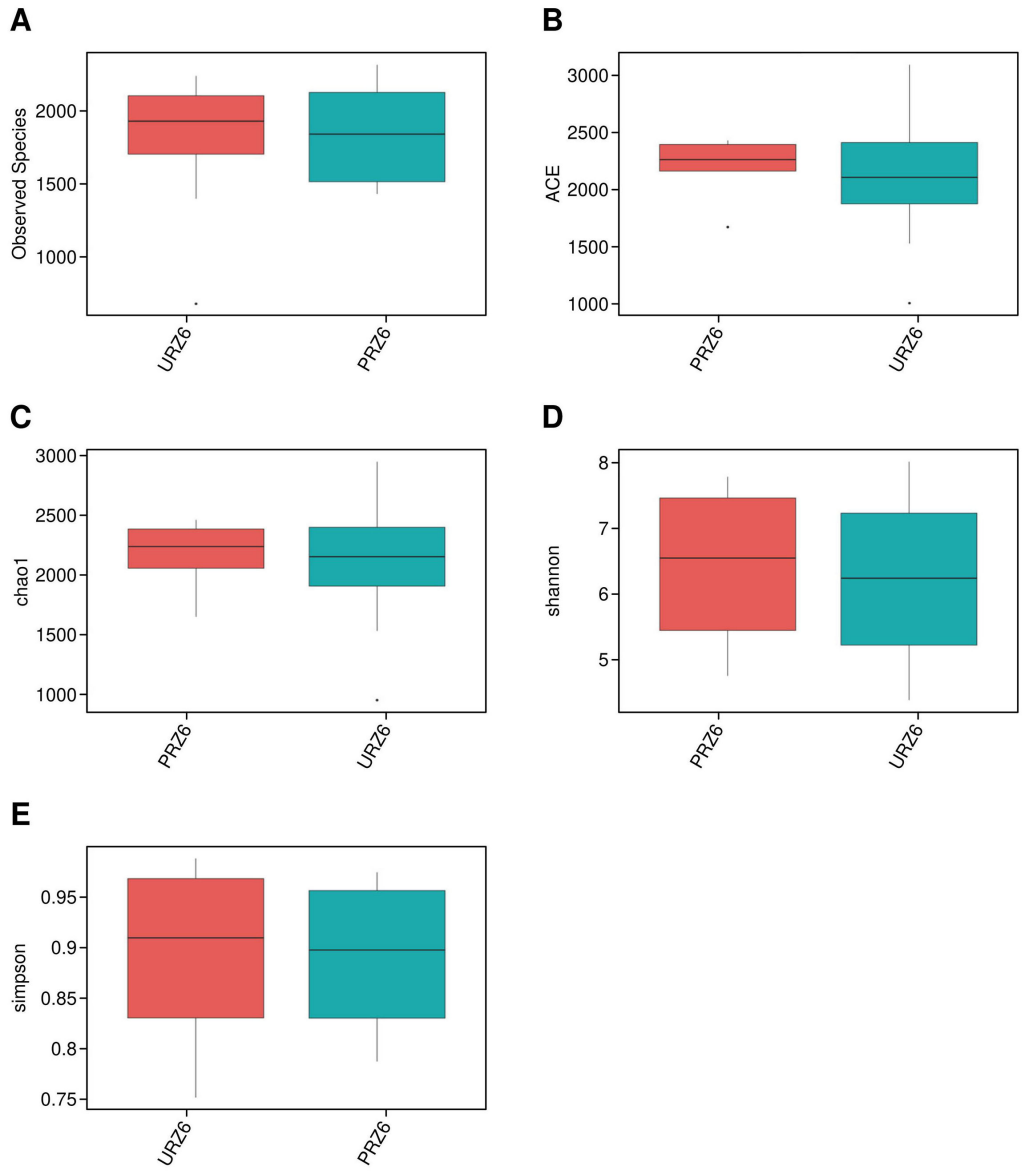
Alpha diversity comparison between URZ6 and PRZ6 groups. **(A–E)** Box plots of observed species, ACE, Chao1, Shannon, and Simpson indices in all groups, respectively.

**Figure 5:**
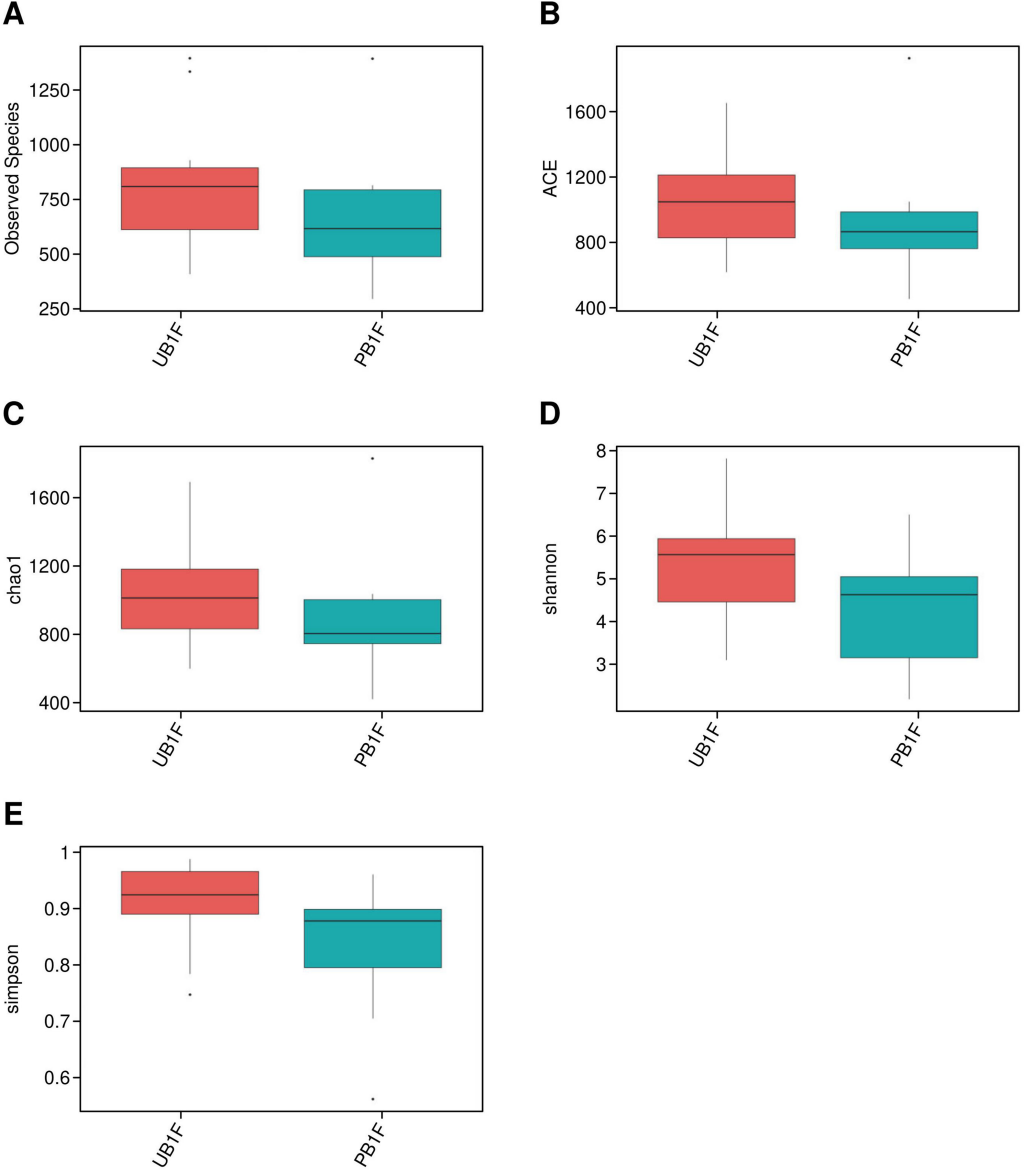
Alpha diversity comparison between UB1F and PB1F groups. **(A–E)** Box plots of observed species, ACE, Chao1, Shannon, and Simpson indices in all groups, respectively.

**Figure 6:**
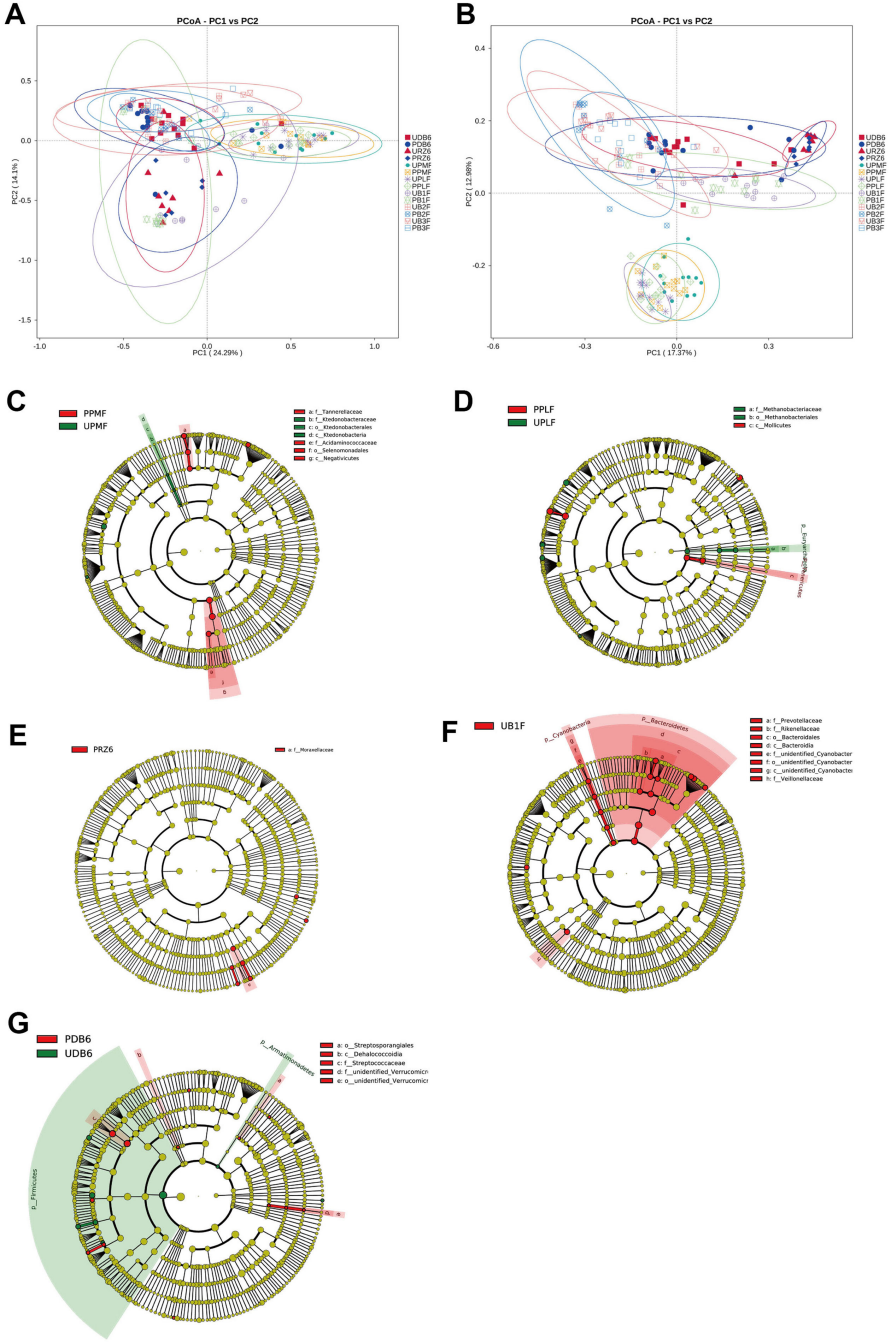
Beta diversity analysis of microbiota communities. **(A)** Principal component analysis (PcoA) uses weighted UniFrac distance. **(B)** PcoA by using unweighted UniFrac distance. **(C–G)** PPMF vs. UPMF, PPLF vs. UPLF, PRZ6 vs. URZ6, PDB6 vs. UDB6, and PB1F vs. UB1F, respectively.

Alpha diversity was assessed using five indices: observed species, ACE, Chao1, Shannon, and Simpson (**Figure 2**, **Table 2**). ANOVA revealed significant differences among groups, with the observed species index lowest in PB2F/UB2F and PB3F/UB3F and highest in URZ6/PRZ6. However, Wilcoxon tests comparing probiotic and control groups (UPLF vs. PPLF, URZ6 vs. PRZ6, UB1F vs. PB1F; **Figures 3**–**5**) showed no statistically significant differences, indicating that supplementation did not markedly alter overall microbial richness or evenness. The significant differences detected by ANOVA are not directly attributable to the probiotic intervention, but are rather reflective of the expected changes in microbial diversity associated with the developmental stage, which might be the main driven factor of significant differences detected in ANOVA. Observed variations between maternal and infant samples were unlikely attribute *delivery* to sequencing depth or batch effects, as rarefaction analysis confirmed comparable coverage and library preparation conditions were identical across all samples, suggesting genuine biological differences consistent with developmental shifts in early-life gut microbiota. Beta diversity analysis at the OTU level using weighted and unweighted UniFrac distances revealed modest but statistically supported separation between groups (**Figures 6A, B**). Weighted UniFrac explained 24.29% and 14.1% of the variation, while unweighted UniFrac explained 17.37% and 12.98%. PERMANOVA confirmed these clustering patterns (weighted R² = 0.126, *p* = 0.032; unweighted R² = 0.109, *p* = 0.041), indicating that probiotic supplementation exerted a subtle but significant effect on overall community structure. Late postpartum samples, including maternal milk samples (URZ6, PRZ6) and infant fecal samples at 6 months (UDB6, PDB6), clustered primarily according to age-related maturation and lactational stage–associated microbial shifts. To avoid ambiguity, we note that URZ6 and PRZ6 represent milk samples, not fecal samples. Therefore, clustering patterns observed for these groups correspond to maturation of the milk microbiome during late lactation. Differential abundance between groups (PPMF vs. UPMF, PPLF vs. UPLF, PRZ6 vs. URZ6, PDB6 vs. UDB6, PB1F vs. UB1F) was initially evaluated using LEfSe (LDA score > 2.0) with Benjamini–Hochberg FDR correction (*q* < 0.05) to reduce false positives (**Figures 6C–G**). Functional predictions from PICRUSt2 were similarly filtered for KEGG pathways with FDR-adjusted *p* < 0.05, ensuring robust identification of putative microbial functions. To account for repeated measures in the longitudinal design, genus-level differences were further validated using MaAsLin2, applying subject ID as a random effect and time point and treatment as fixed effects. After FDR correction (*q* < 0.05), six genera, including *Bifidobacterium*, *Faecalibacterium*, and *Akkermansia*, demonstrated significant time-dependent modulation associated with probiotic supplementation. These results corroborate LEfSe findings while providing a statistically rigorous framework that accounts for intra-subject variability. These analyses indicate that while alpha diversity remained largely unchanged, probiotic supplementation induced subtle but statistically supported shifts in microbial composition over time, and maternal and infant gut communities exhibit biologically meaningful developmental trajectories.

**Table 2:** Alpha diversity indices sorted by observed species index.

Group name	Observed species	ACE	Shannon	Simpson	Chao1
PB2F	266	373.189	3.269	0.736	355.558
UB2F	279	416.323	2.995	0.696	402.778
PB3F	322	463.238	3.888	0.823	445.687
UB3F	348	467.356	4.022	0.838	447.477
UDB6	498	776.791	3.888	0.82	741.132
PDB6	554	892.756	3.646	0.779	853.147
UPLF	593	722.143	6.064	0.958	721.558
PPLF	597	730.45	6.109	0.961	724.474
PPMF	610	769.751	5.812	0.93	768.166
PB1F	656	889.695	4.456	0.83	865.514
UPMF	663	849.604	5.767	0.934	835.205
UB1F	810	1058.903	5.483	0.899	1041.983
URZ6	1728	2074.48	6.232	0.894	2045.562
PRZ6	1794	2184.208	6.456	0.89	2140.91

### 3.4 WGCNA analysis of 16S OTU data

**Figure 7:**
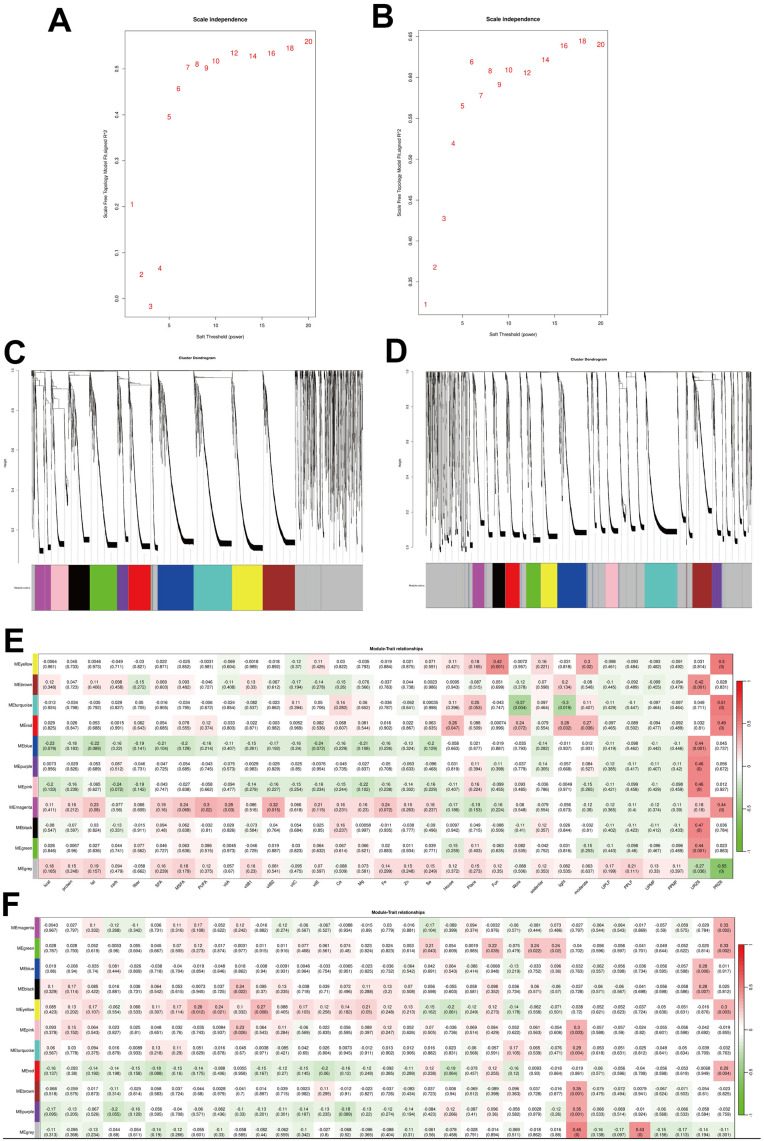
Weighted gene co-expression network analysis. **(A, B)** Soft-thresholding plot of adult **(A)** and newborn **(B)** datasets. **(C, D)** Hierarchical clustering dendrogram of modules in adult **(C)** and infant **(D)** datasets. **(E, F)** Module-trait correlation heatmap of adult **(E)** and newborn **(F)** datasets.

**Figure 8:**
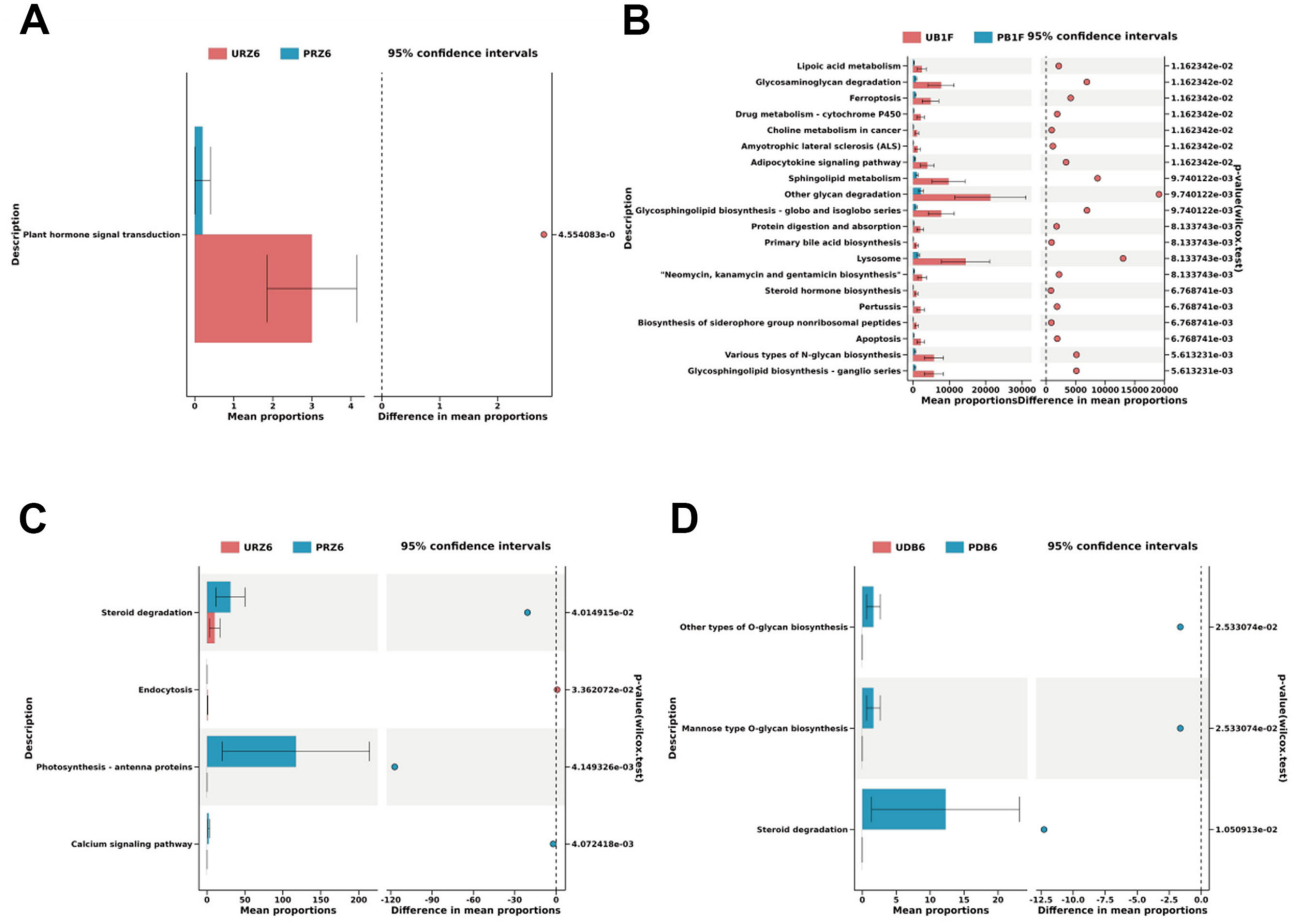
Pathway differences were predicted using PICRUSt2 and represent inferred, not experimentally validated, functional profiles. **(A–C)** KEGG terms significantly different between URZ6 and PRZ6 groups in blue module and magenta module of adult dataset. **(B–D)** KEGG terms significantly different between UDB6 and PDB6 groups in purple and yellow modules of infant dataset.

**Figure 9:**
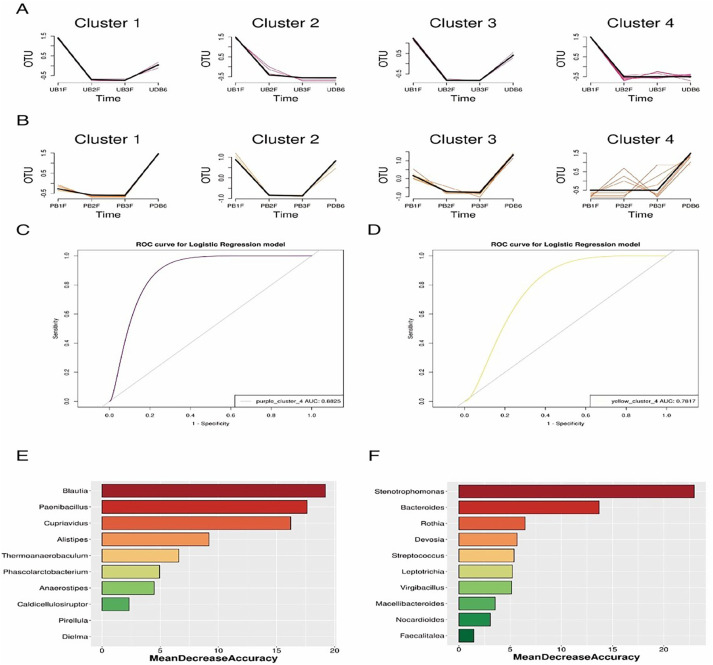
Clusters were annotated with dominant genera and their putative biological functions in gut immune and metabolic development, including early colonizers (*Bifidobacterium*, *Lactobacillus*), short-chain fatty acid producers (*Faecalibacterium*, *Roseburia*), and mucin degraders (*Akkermansia*). **(A)** SourceTracker analysis depicting proportional maternal microbial contribution to infant gut communities at birth and six months postpartum. **(B)** Venn diagram showing shared ASVs between maternal and infant samples across probiotic and control groups. **(C, D)** represent the top 8 and 10 mean decrease accuracy genera using random forest model analysis in cluster 4 of the purple and yellow modules in the infant dataset, respectively. **(E, F)** ROC curves were generated using 10-fold cross-validation and independently validated on a 20% test subset to ensure model generalizability and prevent overfitting.

We utilized Weighted Gene Co-expression Network Analysis (WGCNA) to construct a correlation network, examining pairwise correlations between OTUs across adult and infant groups based on relative OTU abundance. This approach effectively analyzes complex microbiome communities, allowing us to explore associations between specific phenotypes or diseases and identify target genes and networks for further investigation. We divided the OTUs into adult and infant datasets. The adult dataset comprised the PPMF, UPMF, PPLF, UPLF, PRZ6, and URZ6 groups, while the infant dataset included PB1F, UB1F, PB2F, UB2F, PB3F, UB3F, PDB6, and UDB6 groups. The 8,000 most variable OTUs were extracted based on median absolute deviation, and their correlation values were computed to define the OTU co-expression network. The OTU abundance similarity matrix was converted into an adjacency matrix to establish connections based on scale-free topology. In the adult dataset, a soft thresholding power of β = 16 (scale-free R² = 0.53) was chosen to ensure a scale-free network (**Figure 7A**), while the same power was applied to the infant dataset (scale-free R² = 0.64) (**Figure 7B**). Using the dynamic tree-cut method, we identified co-occurring OTU modules, merging modules with over 80% similarity. This resulted in 11 distinct modules for both the adult and infant datasets. In the adult dataset, module sizes ranged from a minimum of 150 OTUs in the purple module to 518 OTUs in the turquoise module (**Figure 7C**). A total of 1,007 OTUs were unclassified and categorized into the grey module. In the infant dataset, module sizes varied from 108 OTUs in the purple module to 370 OTUs in the turquoise module, with 1,649 OTUs remaining unclassified in the grey module (**Table 3**). The OTU network, alongside the hierarchical clustering dendrogram and modules, is depicted in **Figure 7D**. We collected data on standard energy and nutrient indices, exercise duration, and microelements for trait analysis from pregnant women. This information was transformed into traits to identify the most relevant modules for each group. The correlation between traits and module eigengenes was summarized in a heatmap (**Figures 7E, F**). Several modules demonstrated significant correlations with various characteristics. In the adult dataset, the blue module exhibited negative correlations with multiple nutrient traits (e.g., kcal, protein, fat, fiber, SFA, MUFA, PUFA, vitamin E, and Mg). Still, it was positively correlated with the URZ6 group, suggesting the blue module contains principal microbiota. Conversely, the magenta module positively associated with nutrient traits such as fat, PUFA, MSFA, vitamin A, vitamin B2, and iron, correlating with the PRZ6 group. These two modules exhibited opposite correlations with nutrient traits (**Figure 7E**). We employed PICRUSt2 to predict pathways associated with these modules. The magenta module showed significant differences in four KEGG terms between URZ6 and PRZ6: calcium signaling pathway, endocytosis, photosynthesis-antenna proteins, and steroid degradation (**Figures 8A, B**). In the infant dataset, two notable modules were identified. The purple module correlated positively with U1BF but negatively with several maternal nutrient indices (carbohydrates, fiber, Mg, and vitamin E). The yellow module was positively correlated with PDB6 and several maternal nutrient indices (PUFA, vitamin A, vitamin B2, and iron), indicating that these modules may represent the dominant gut microbiota at birth and six months postpartum (**Figure 7F**). PICRUSt2 analysis revealed 184 significantly different KEGG terms between U1BF and P1BF in the purple module, highlighting key processes such as glycosphingolipid biosynthesis and protein digestion. The yellow module displayed three significant terms differentiating UDB6 and PDB6, including mannose-type O-glycan biosynthesis and steroid degradation (**Figures 8C, D**). Functional inference using PICRUSt2 predicted differences in several KEGG pathways between groups, including calcium signaling, endocytosis, and steroid degradation (**Figures 8A–D**). However, as PICRUSt2 estimates functional potential based on 16S rRNA-derived taxonomic profiles, these results should be interpreted as predictive rather than confirmatory. Further validation through shotgun metagenomic or metabolomic analyses is required to substantiate these inferred functional shifts. Time-series clustering using *mfuzz* revealed four distinct temporal patterns of OTU abundance (**Figures 9A, B**). Cluster 1 (predominantly *Bifidobacterium* and *Lactobacillus*) was enriched in early-infant samples, consistent with their roles in carbohydrate metabolism and immune tolerance induction during early colonization. Cluster 2 (*Faecalibacterium*, *Roseburia*) increased over time, reflecting the establishment of butyrate-producing commensals associated with gut barrier maturation. Cluster 3 (*Akkermansia* and *Ruminococcus*) showed moderate enrichment at six months postpartum, suggesting involvement in mucin degradation and host–microbe interface regulation. Cluster 4 (*Escherichia/Shigella*) displayed transient abundance in early samples, indicative of facultative anaerobe dominance prior to stable colonization. These temporal trajectories collectively illustrate a dynamic succession from pioneer taxa to mature anaerobes, paralleling infant gut immune and metabolic development (**Figures 9A, B**). To minimize the risk of overfitting in the classification of key genera associated with U1BF and PDB6 groups (**Figures 9C, D**), model performance was re-evaluated using rigorous validation protocols. Random forest models were subjected to 10-fold cross-validation, and model robustness was further confirmed by an 80/20 train–test split procedure. The mean cross-validated AUCs were 0.86 ± 0.04 for the purple module (U1BF classifier) and 0.75 ± 0.05 for the yellow module (PDB6 classifier), closely matching the original AUCs (0.88 and 0.78, respectively). These results confirm that classifier performance was not driven by overfitting but reflects genuine discriminatory power of the identified genera (**Figures 9C, D**). SourceTracker2 was employed to estimate the contribution of maternal gut microbiota to the infant microbiome composition. On average, 26.4% of the infant gut microbiota at birth and 18.7% at six months postpartum were traceable to the maternal microbiota. Notably, infants from probiotic-supplemented mothers (PB1F, PDB6) exhibited a higher proportion of shared amplicon sequence variants (ASVs) with maternal samples (mean 32.1%) compared with controls (mean 21.4%, p = 0.038). The most commonly shared genera included Bifidobacterium, Lactobacillus, and Akkermansia, suggesting that maternal probiotic intake may facilitate vertical transmission or selective enrichment of beneficial taxa in infants. ROC curve analysis assessed the performance of candidate biomarkers, with the top eight genera from cluster 4 of the purple module yielding an area under the ROC curve (AUC) of 0.88 for U1BF (**Figure 9E**), and the top ten genera from the yellow module producing an AUC of 0.78 for PDB6 (**Figure 9F**). Although no statistically significant differences in overall nutrient intake were detected between probiotic and non-probiotic groups based on FFQ analysis, WGCNA revealed correlations between microbial modules and continuous nutrient variables. This apparent discrepancy arises because the FFQ captures macronutrient and micronutrient intake at a group level, whereas WGCNA identifies covariance patterns at the individual level. Thus, even in the absence of between-group dietary differences, microbiota composition may still covary with subtle intra-individual differences in dietary profiles. These findings suggest that the associations identified through WGCNA reflect biological coupling between diet and microbial structure, rather than direct effects of dietary intervention.

**Table 3:** Number of OTUs in each module of the adult and infant datasets.

Module	Number of OTUs	Dataset
Purple	150	Adult
Magenta	208	Adult
Pink	235	Adult
Black	280	Adult
Red	292	Adult
Green	362	Adult
Yellow	402	Adult
Brown	424	Adult
Blue	474	Adult
Turquoise	518	adult
Gray	1007	adult
Purple	108	infant
Magenta	133	infant
Black	145	infant
Pink	145	infant
Green	157	infant
Red	157	infant
Yellow	188	infant
Brown	222	infant
Blue	352	infant
Turquoise	370	infant
Gray	1649	infant

## 4 Discussion

Probiotics are widely recognized for their potential health benefits (Milani et al., 2017). Numerous clinical studies have highlighted their effectiveness in treating various conditions, including necrotizing enteritis, alleviating hernia-related pain, reducing diarrhea duration in children, improving symptoms of irritable bowel syndrome, and mitigating antibiotic-associated diarrhea (Milani et al., 2017). While the impact of probiotics has been studied extensively in non-pregnant women, research specifically addressing their effects during pregnancy remains limited (Hui et al., 2021). Probiotic supplementation during pregnancy has been proposed to influence both the gut and vaginal microbiomes by altering microbial community structures (Gohir et al., 2015). Some studies suggest that increasing the abundance of specific genera, such as Bifidobacterium or Lactobacillus, may help treat certain conditions or enhance health (Matharu et al., 2022). However, this remains a topic of debate, as other research indicates that probiotics may not significantly alter microbiota’s alpha and beta diversity (Backhed et al., 2015). This discrepancy may arise from the notion that probiotics do not directly influence gut microbiota composition but instead modulate the gut environment through various pathways, potentially leading to structural changes in the microbiota. Historically, it was believed that fetuses lacked intestinal microbiota and that the meconium was entirely sterile (Milani et al., 2017). Recent evidence, however, suggests that fetal gut microbiota colonization may begin earlier than previously thought, potentially influenced by maternal microbiota (Coscia et al., 2021). Despite these advances, direct evidence for gut microbiota colonization in fetuses remains elusive.

Multiple factors, including mode of delivery, maternal microbiota, environmental influences, health conditions, and antibiotic use, shape the gut microbiota of infants (Jeong, 2022). The feeding method is particularly crucial as breast milk is the primary nutrition source for infants, with its microbiome evolving during lactation. Notably, studies have shown that breastfed infants’ gut microbiota significantly differs from formula-fed infants. The relationship between maternal microbiota structure and infant microbiota development and the potential long-term implications of perinatal conditions on microbiota composition in adulthood warrant further investigation. Given the possible link between maternal and infant gut microbiota, we hypothesized that probiotic supplementation could influence the microbiota structure of pregnant women and subsequently affect their infants’ gut microbiota. We selected *Bifidobacterium longum*, *Lactobacillus bulgaricus*, and *Streptococcus thermophilus* as the probiotics for this study. After administering probiotics, we collected fecal samples before delivery and milk samples at six months postpartum. Our investigation initially focused on the effects of probiotics on maternal nutrient status; however, we observed no statistically significant differences in nutrient intake between the probiotic and non-probiotic groups. We then explored the potential influence of gut microbiota on maternal nutrient status. 16S rRNA gene sequencing allowed us to profile the gut and milk microbiota of UPMF, PPMF, UPLF, and PPLF. Although the alpha diversity indices did not show significant alterations, the beta diversity analysis revealed an overrepresentation of several genera in PPLF compared to UPLF, including *Gemella*, *Methanobrevibacter*, *Shuttleworthia*, *Staphylococcus*, *Blautia*, and *Veillonella*. Notably, *Blautia* has been linked to visceral fat accumulation in adults (Nuriel-Ohayon et al., 2016), and *Veillonella*, a core gut genus, was upregulated by probiotics (Oikonomou et al., 2020). These microbiotas changes observed in our study may be critical for nutritional metabolism during pregnancy, despite no changes in the overall richness and diversity of the maternal gut microbiota. Our source-tracking analysis supports partial vertical microbial transmission from mothers to infants, consistent with recent reports demonstrating that maternal gut and breast milk microbiota contribute to neonatal colonization. The increased proportion of shared ASVs in probiotic-supplemented dyads suggests that probiotic intake during pregnancy may enhance transmission of beneficial commensals, such as *Bifidobacterium* and *Lactobacillus*, to the infant gut. These findings highlight a plausible mechanistic link between maternal supplementation and early-life microbial establishment, warranting further validation through metagenomic tracking and strain-level analysis.

We employed WGCNA to analyze adult samples and construct a network of all genera within the maternal gut microbiota. The module trait relationship analysis identified two key modules associated with nutrient status. The blue module demonstrated negative correlations with kcal, protein, fat, fiber, SFA, MUFA, PUFA, vitamin E, and Mg. In contrast, the magenta module positively correlated with fat, PUFA, MSFA, vitamin A, B2, and iron. Furthermore, these modules exhibited opposing correlations with the milk microbiota profiles (URZ6 and PRZ6) collected at 6 months postpartum indicating they may represent core genera in these populations. Subsequent functional enrichment analysis using PICRUST2 revealed that the blue module had only one significantly abundant KEGG term plant hormone signal transduction in the URZ6 group.

In contrast, the magenta module contained several significant terms in the PRZ6 group, including calcium signaling pathway, steroid degradation, and endocytosis in the URZ6 group. These differential pathways may reflect long-term changes in maternal nutrient status resulting from probiotic supplementation, potentially influencing infant gut microbiota through breastfeeding. Focusing on the gut microbiota of infants, it has been traditionally assumed that few bacterial genera inhabit the fetal intestinal tract. However, recent reports indicate that microbial colonization begins prenatally. We sampled infant feces at four time points: delivery day, 3 days, 14 days, and 6 months after birth. Our analysis of the richness of all samples indicated that the observed species index was highest on delivery day and lowest at 3 days postpartum, with values increasing at 14 days and 6 months. This trend suggests a developmental process wherein newborns initially harbor maternal microbiota, which gradually evolves as their intestinal systems adapt to their environments and maternal milk composition. The relatively low observed species index in the PB2F/UB2F and PB3F/UB3F groups may reflect this developmental stage. We found no significant difference in alpha diversity between UB1F and PB1F, and LEfSe analysis identified *Occallatibacter* as the only over-represented genus in this comparison. Limited research exists on *Occallatibacter*, and further studies are needed to clarify its role.

WGCNA of infant samples revealed two distinct modules. The purple module was negatively correlated with numerous nutrient terms (e.g., kcal, protein, fat, fiber, Mg) but positively correlated with the U1BF group. In contrast, the yellow module positively correlated with nutrient terms and the PDB6 group. Functional enrichment analysis via PICRUST2 showed that 20 KEGG terms were over-represented in the purple module for the U1BF group. In contrast, only three KEGG terms in the yellow module were over-represented in the PDB6 group, specifically mannose-type O-glycan biosynthesis, other types of O-glycan biosynthesis, and steroid degradation. The higher number of over-represented KEGG terms in the purple module may be attributed to probiotic supplementation during pregnancy, while the terms in the PDB6 group likely reflect intestinal development and adaptation to milk digestion. The purple and yellow modules exhibited opposing relationships with maternal nutrient factors, demonstrating positive correlations with the first time (birth) and the final time (6 months postpartum), respectively. Consequently, we analyzed these modules’ time series across the four time points. The most abundant clusters from both modules were selected to evaluate potential biomarkers of changes in the infant gut microbiota attributed to probiotic supplementation at delivery and at 6 months postpartum using random forest model analysis and forward stepwise logistic regression. Candidate biomarkers were assessed through ROC curve analysis. Because PICRUSt2 relies on reference genome mapping from 16S data, it infers rather than measure’s metabolic function. Consequently, the observed enrichment in pathways such as steroid degradation or glycosphingolipid biosynthesis should be viewed as hypothesis-generating, not definitive. These predictions provide valuable leads for future functional validation using shotgun metagenomics, metatranscriptomics, or targeted metabolomics, which can more accurately define probiotic-associated metabolic adaptations.

The decreased accuracy genera identified as predictors of U1BF included *Blautia*, *Paenibacillus*, *Cupriavidus*, *Alistipes*, *Thermoanaerobaculum*, *Phascolarctobacterium*, *Anaerostipes*, and *Caldicellulosiruptor*. Notably, *Anaerostipes* is known to convert inositol stereoisomers into propionate and acetate anaerobically, making it vital for host dietary health. Additionally, *Alistipes* have been associated with various diseases, including cardiovascular conditions (Ozato et al., 2019). Its pro-inflammatory lipopolysaccharides may contribute to hypertension by promoting inflammation and altering epithelial integrity, thereby affecting the balance of butyrate-producing bacteria known for their anti-inflammatory properties (Prince et al., 2014). In cluster 4 of the yellow module, the top 10 mean decrease accuracy genera identified as predictors of PDB6 included *Stenotrophomonas*, *Bacteroides*, *Rothia*, *Devosia*, *Streptococcus*, *Leptotrichia*, *Virgibacillus*, *Macellibacteroides*, and *Nocardioides*. These genera, particularly *Bacteroides*, are critical for infant gut development. The composition of *Bacteroides* can vary in the infant gut, significantly influencing nutrient absorption and metabolism during the first year of life (Principi and Esposito, 2016; Sanders, 2016). We propose that probiotic supplementation during late pregnancy positively affects the abundance of *Bacteroides*, potentially benefiting infant gut development and nutrient absorption capabilities. Several limitations should be acknowledged in this study. First, our findings may not be generalizable as a single-center cross-sectional study. A multi-center approach with a larger participant pool would provide more robust insights into trends across diverse maternal populations and additional infant time points. The study was open-label and unblinded, as no placebo was administered to the control group. This design may introduce expectation or reporting bias, particularly affecting subjective data such as the food-frequency questionnaire (FFQ). Future studies using placebo-controlled, double-blind designs are needed to minimize these sources of bias. While we utilized 16S rRNA gene sequencing to study gut microbiota, this method may not provide sufficient resolution for species-level identification or functional predictions. Future research employing metagenomics sequencing would enable more precise species, strain-level analysis, and functional gene and pathway information.

## 5 Conclusion

This study provides an exploratory analysis of maternal and infant gut microbiota in response to probiotic supplementation during pregnancy. While overall alpha diversity and maternal nutrient intake were not significantly altered, specific genera such as *Blautia*, *Methanobrevibacter*, and *Veillonella* were enriched in the probiotic group, suggesting potential metabolic benefits. Infant gut microbiota development followed expected postnatal trajectories, with observed species indices increasing over time and correlating with maternal microbial modules. These findings highlight associative links between maternal supplementation and early-life microbiota composition but require validation in larger, multi-center cohorts and through functional and mechanistic studies to substantiate potential health effects.

## Data Availability

The data presented in the study are deposited in the China Nucleotide Sequence Archive (CNSA: https://db.cngb.org/cnsa) under accession code CNP0000301.
