# Supplementary material for: Probiotic modulation of maternal gut and milk microbiota and potential implications for infant microbial development in the perinatal period
Source: Front Cell Infect Microbiol. 2025 Dec 11;15:1715989. doi: 10.3389/fcimb.2025.1715989 (PMC12738936; doi:10.3389/fcimb.2025.1715989)
Supplement: Supplementary file 1 [file SupplementaryFile1.docx]

**Supplementary Files**


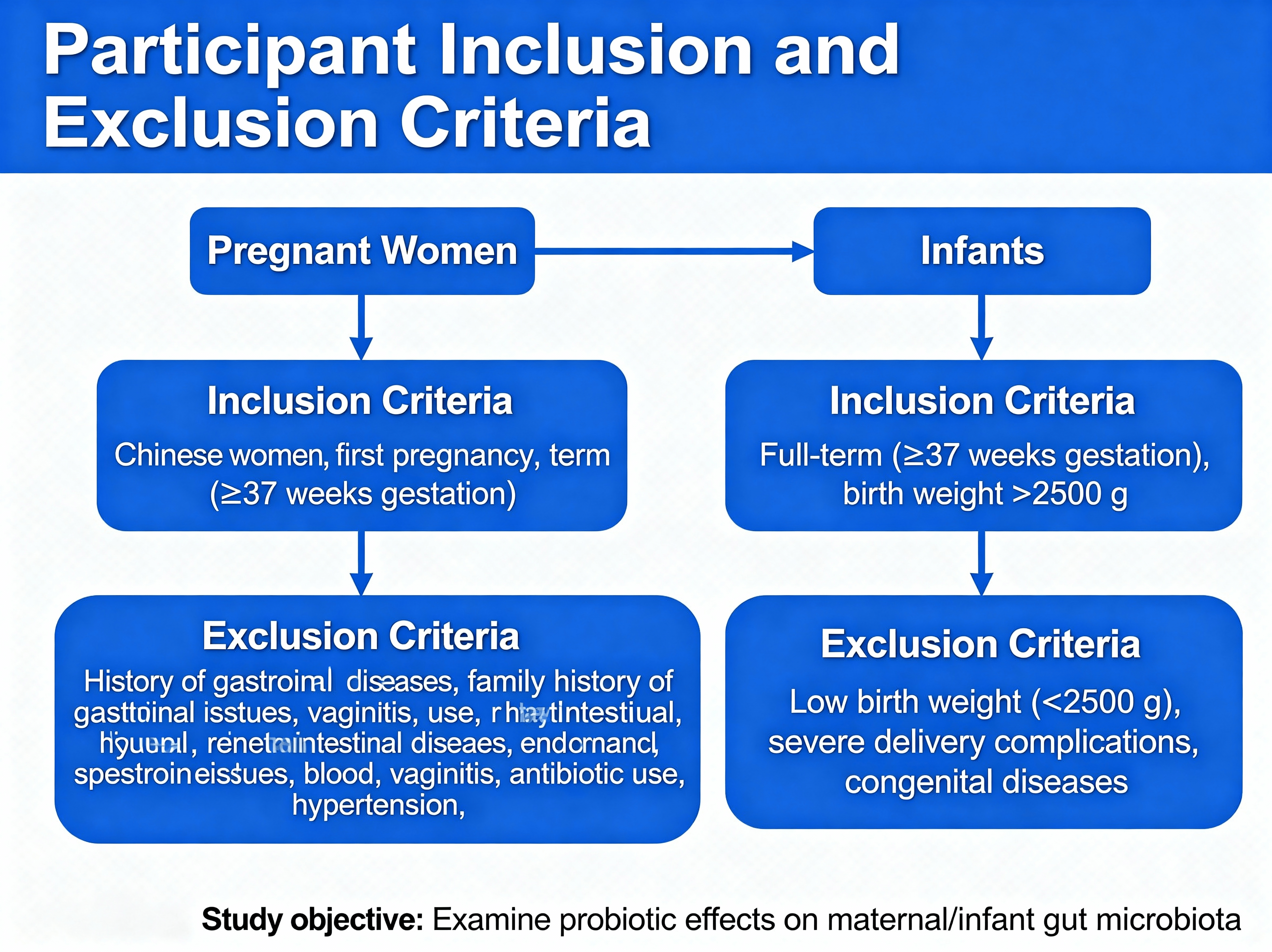


**Figure S1: Flowchart of participant enrollment, eligibility screening, group allocation, and exclusion criteria for the study cohort**
